# Implementing the Baby One Program: a qualitative evaluation of family-centred child health promotion in remote Australian Aboriginal communities

**DOI:** 10.1186/s12884-018-1711-7

**Published:** 2018-03-24

**Authors:** Sandra Campbell, Janya McCalman, Michelle Redman-MacLaren, Karla Canuto, Kristina Vine, Jenny Sewter, Malcolm McDonald

**Affiliations:** 1 Centre for Chronic Disease Prevention, College of Public Health, Medical & Veterinary Sciences, James Cook University, PO Box 6811, Cairns, QLD 4870 Australia; 2 Centre for Indigenous Health Equity Research, School of Health, Medical and Applied Sciences, Central Queensland University, PO Box 7815, Cairns, QLD 4870 Australia; 3 The Cairns Institute, James Cook University, PO Box 6811, Cairns, QLD 4870 Australia; 4 College of Medicine and Dentistry, James Cook University, PO Box 6811, Cairns, QLD 4870 Australia; 5 Apunipima Cape York Health Council, PO Box 12045, Westcourt, Cairns, QLD 4870 Australia

**Keywords:** Child health, Maternal health, Pregnancy, Aboriginal, Torres Strait Islander, Indigenous, Health promotion, Cape York, Health services

## Abstract

**Background:**

A healthy start predicts better health in later life. Many remote-living Aboriginal and Torres Strait Islander Australian families lack access to consistent, culturally-safe health services. This paper presents a study of implementation of the Baby One Program (BOP). The BOP was designed as a family-centred, Indigenous Healthworker-led, home-visiting model of care focused on promoting family health to give children the best start to life. It was developed by Aboriginal community-controlled Apunipima Cape York Health Council and delivered in Queensland Cape York remote communities. We aimed to determine how the BOP was implemented, enablers, strategies used and formative implementation outcomes.

**Methods:**

The qualitative approach utilised theoretical and purposive sampling to explore people’s experiences of a program implementation process. Data were generated from semi-structured interviews with four family members enrolled in the BOP and 24 Apunipima staff members. In addition, twenty community members, including two program users, participated in a men’s community focus group. The findings are presented according to themes arising from the data.

**Results:**

The BOP was rolled out in nine remote Cape York communities between July 2014 and December 2015 and there was high uptake. Indigenous Healthworkers were supported by midwives and maternal and child health nurses to deliver health education to 161 eligible families. The key to effective implementation of family-centred care appeared to be the relationships formed between health practitioners, especially Indigenous Healthworkers, and families. The data revealed the following themes: challenging environments for new families and valuing cultural ways, resourcing program delivery, working towards a team approach, negotiating the cultural interface, engaging families, exchanging knowledge through ‘yarning’, strengthening the workforce, and seeing health changes in families. Healthworker education and training, and knowledge exchange between Healthworkers, midwives and nurses was critical to program effectiveness. The program continues to grow despite substantial logistic, financial and practical challenges.

**Conclusions:**

This study describes an evolving process and explores how health providers connect with families and how the program responds to family and cultural issues. Program development is ongoing; strengthened by more community-level involvement, embedded strategies for ongoing self-evaluation and continuous quality improvements that are responsive to family needs.

**Electronic supplementary material:**

The online version of this article (10.1186/s12884-018-1711-7) contains supplementary material, which is available to authorized users.

## Background

A healthy start predicts a healthy life [1]. A child’s first 1000 days determine brain development, attachment, risk of chronic disease and other physiological, mental and social outcomes, probably across generations [1, 2]. Regular health service contact should improve health outcomes through early detection and management of disease precursors, and opportunities for disease prevention.

Effective antenatal care can make a critical contribution by addressing health literacy and parenting behaviours, with a focus on nutrition, alcohol and smoking cessation and the benefits of breastfeeding. However, for remote-living Aboriginal Australian and Torres Strait Islander (hereafter Indigenous) families, there are major geographical, environmental, economic, cultural and social challenges. Many families lack ready access to consistent, culturally-safe maternal and child health services, and health promoting educational resources [3, 4].

In some high-income countries, disparities in maternal and child health outcomes of Indigenous populations have motivated development of community-based family-centred healthcare [5, 6]. Family-centred care is ‘a way of caring for children and their families within health services which ensures that care is planned around the whole family…all family members are recognised as care recipients’ [7]. Care is based on six key elements: [8](i) ‘Recognizing the family as central to and/or the constant in the child’s life, and the child’s primary source of strength and support’, (ii) ‘Acknowledging the uniqueness and diversity of children and families’ (iii) ‘Acknowledging that parents bring expertise to both the individual care-giving level and the systems level’ (iv) ‘Recognizing that family-centred care is competency enhancing rather than weakness focused’ (v) ‘Encouraging the development of true collaborative relationships between families and health-care providers, and partnership’, and (vi), ‘Facilitating family-to-family support and networking , and providing services that provide emotional and financial support to meet the needs of families’.

Internationally, family-centred interventions for Indigenous families have produced positive health outcomes for children, parents and other primary carers, but these can take years to manifest. The Family Spirit intervention delivered by community-based paraprofessionals for North American First Nations families was developed through participatory research over 10 years from 1995 [5]. Once implemented, a randomised controlled trial in 2015 assessed maternal and child emotional and behavioural outcomes 36 months after birth. Children of families who received the intervention had fewer social and emotional and behavioural problems. Their mothers had significantly greater parenting knowledge, parental locus of control, fewer depressive problems, less stress, and a lower past month use of marijuana and illegal drugs compared to the control group [8, 9].

This study presents a qualitative evaluation of the initial 18 months implementation of an Australian family-centred program for improving child health. The Baby One Program (BOP) was developed by Apunipima Cape York Health Council (hereafter Apunipima) and is delivered in Queensland’s remote Cape York Indigenous communities. The vision of the program is to improve long-term health by ‘giving children the best start to a healthy life’. The aim of this study was, to determine how the BOP was implemented. The key research questions were: (i) What were the enablers and strategies used in implementing the BOP? and (ii) What were the formative implementation outcomes?

## Methods

### The setting

**Fig. 1:**
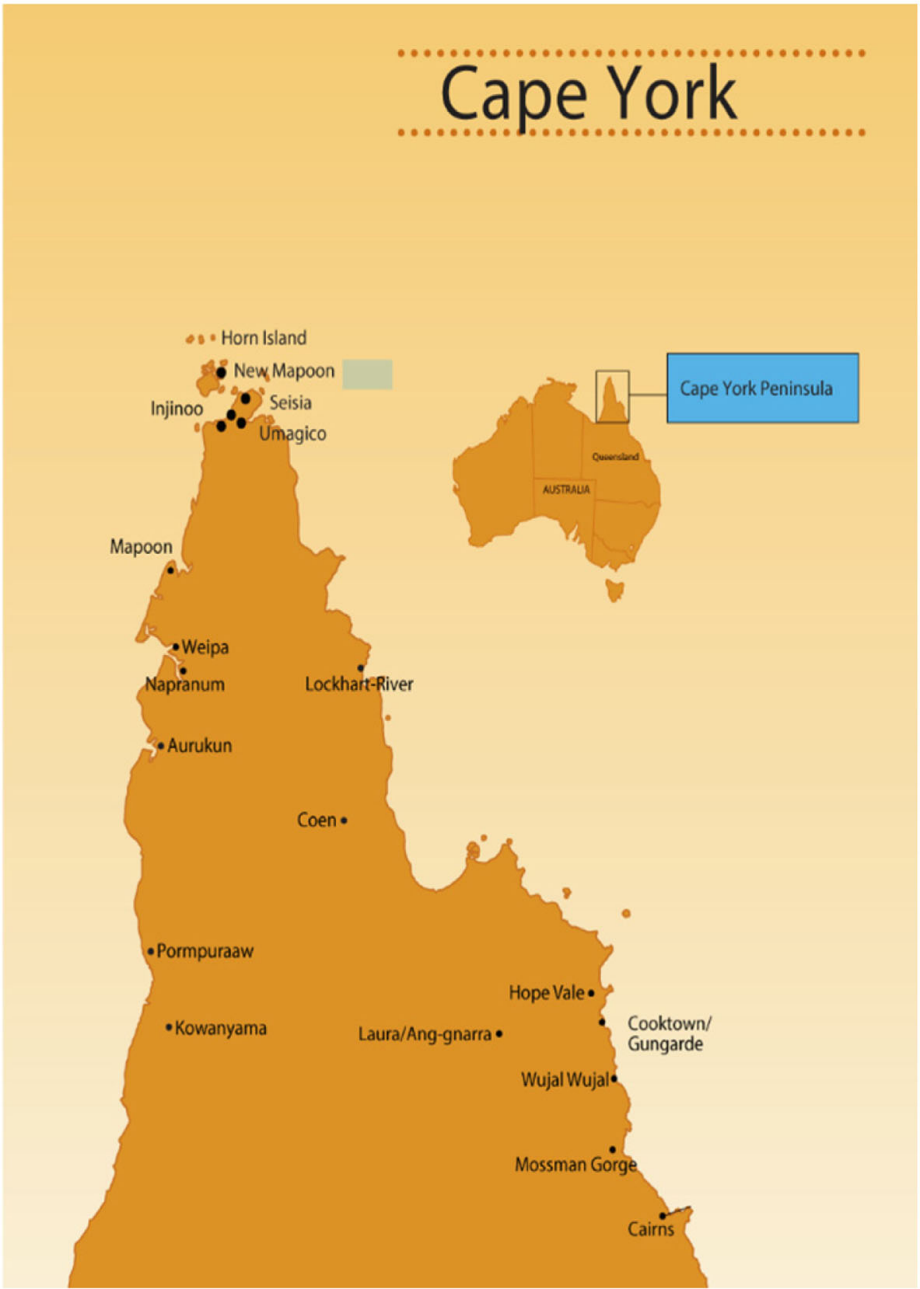
Map of Cape York region (Source: Apunipima Cape York Health Council)

Cape York covers an area of 137,000 km^2^, about the landmass of England. The population is approximately 18,000, 60% of whom are Indigenous Australians [10], dispersed across a network of small communities and towns (Fig 1).

Most Aboriginal communities in the region have been, at one time, dispossessed and brutalised by outside colonisation. Today, limited employment opportunities contribute to socioeconomic disadvantage, the primary driver of the high maternal and neonatal morbidity and mortality when compared to the non-Indigenous population [11].

Health services are provided by state government Queensland Health, the Royal Flying Doctor Service, and Apunipima. Apunipima is an Aboriginal community-controlled health organisation that delivers primary health services to 11 Cape York communities. In the region, Cooktown Hospital only accepts low-risk pregnancies whilst the hospital at Weipa provides no peri-partum obstetric services. Thus, at 36 weeks gestation most pregnant Cape York Indigenous women must journey south to Cairns to await childbirth.

### Development and implementation of the Baby One Program

In Cape York communities, when a new baby is born, he or she is often referred to as ‘Baby One’. The BOP was introduced on 1 July 2014 designed as a structured, Indigenous Healthworker-led family visiting program that begins at confirmation of pregnancy and continues until ‘Baby One’ reaches 2 years and 10 months of age. Completion of program delivery is deliberately timed for assessment of family engagement with local primary health services; based on whether the family attends the child’s three-year health check.

The program was designed for Indigenous Healthworkers to engage with families and infants across 15 visits throughout pregnancy and the early childhood years. Visits were delivered in a place preferred by the family – usually outside a clinical setting. Timing of the visits was intended to complement an existing clinical schedule, so Healthworkers, midwives and child health nurses could work in partnership. Indigenous Healthworkers engaged with families using a case-load model in collaboration with midwives and child health nurses. There was potential for the Healthworker and family to develop a strong relationship over several years of continuous care.

**Fig. 2:**
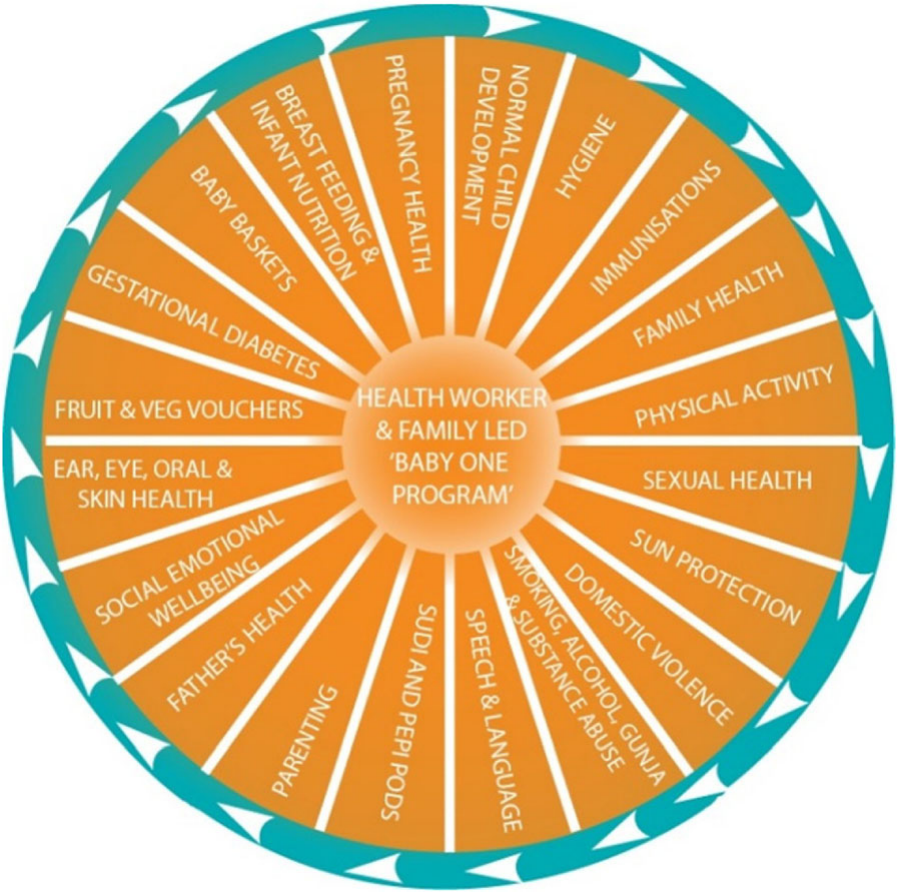
The Apunipima Baby One Program model (Source: Apunipima Cape York Health Council)

BOP components included seven baby baskets and relevant health promotion ‘yarning’ (conversation) topics and activities. Basket contents were used to support key child and maternal health promotion messages and included items such as clothing, information sheets, recipe books, personal hygiene items for the mother and a Pēpi-Pod® Program portable sleep space accompanied by safe sleep education. Thirty-seven yarning topics focus on the issues outlined in Fig. 2.

The BOP was implemented using a pragmatic, stepwise approach driven by each community’s primary health service capacity and staffing arrangements. Delivery was initially in four of the 11 Cape York communities and was progressively extended to nine communities by December 2015. Between July 1, 2014 and December 30, 2015, the BOP enrolled 161 pregnant women and their families. There was 100% uptake of the program; all families who were invited to enrol in the BOP model of care agreed to participate.

### Choice of study method and ethics

A qualitative approach used semi-structured interviews and a focus group to explore experiences of people involved in implementation and also the participants in the BOP. The James Cook University Human Research Ethics Committee granted ethics approval (H6260). Apunipima commissioned the study through James Cook University. Interview participants gave written informed consent and focus group participants verbal consent.

### Data and analysis

The process started with face-to-face interviews with 24 Apunipima staff (Table 1); BOP Indigenous Healthworkers, Men’s Healthworkers, other health practitioners (nurses, midwives, medical practitioners, allied health staff, health promotion officers) and managerial and administrative staff members (Additional files 1 and 2). These participants were directly involved in the program development, program delivery or provided related services or program support. BOP Healthworkers were interviewed by Indigenous Apunipima staff members. The remaining interviews were researcher-led (one Indigenous, one non-Indigenous), guided by semi-structured schedules. Participants responded to a series of specific questions about family visits, yarning topics, engagement, family-centredness, Healthworker training and leadership, professional support for Healthworkers and the most significant changes they observed. Researchers employed tenets of grounded theory [12, 13] to direct interview data collection; the focus of interview questions evolved to explore emerging issues. Audio recording malfunctions occurred during three one-to-one interviews and a men’s Healthworker focus group with four participants. One interview was repeated. Dot point summaries collated by the interviewers were substituted for the remaining interviews and focus group.

**Table 1:** Research participants

Participant type	Participants	Indigenous	Female	Male
Baby One Program family members (Interviews)	4	4 (100%)	3 (50%)	1 (50%)
Other family and community members (Men’s focus group including 2 BOP users)	20	20 (100%)	0	20 (100%)
Healthworkers	6	6 (100%)	6 (100%)	0 (0%)
Other Apunipima staff members	18	10 (56%)	12 (67%)	6 (33%)
Total	48	40 (83%)	21 (44%)	27 (56%)

In the second phase of data collection, Indigenous Apunipima staff invited family participants to face-to-face interviews (Additional file 3). Ideally, family members would have participated in their home community and been recruited and interviewed in the participant’s first language or language of choice. Necessity driven by scarce resources meant interviews were conducted in English while expectant mothers were in Cairns awaiting childbirth, a potentially stressful period due to dislocation from community and loved ones. Four family members participated (two mothers and a couple). The interviews focused on experiences of parenting; participants were asked about aspects of the program that worked well and those that did not work well, specifically (i) baby baskets and health information; (ii) delivery of the program by Indigenous Healthworkers; (iii) support when women came to Cairns to give birth; and (iv) program engagement with family members. All interviews were arranged at a time convenient to participants late in 2015.

Final data collection came from a focus group attended by 20 men (including two men from families enrolled in the BOP program), led by Apunipima’s Men’s Health Team (Indigenous male Healthworkers) convened in a Cape York community where the BOP was implemented.

Data from interviews with Apunipima staff were coded and compared until recurrent concepts and interrelationships were identified. The process was repeated until higher order constructs and relationships could be modelled to explain the data, forming a theoretical model of BOP implementation. The theoretical model was presented to staff and the Research Governance Committee at Apunipima. However, presentation of the data in a theoretical model was not supported as being an accurate reflection of their experience. Strong views were expressed that while the model may have been consistent with early development of the program, it was no longer relevant or applicable to current practice. The research data was pertinent to time from the outset of BOP implementation, however, the program transformed across 18 months of development. In this instance, subjectivity inherent in grounded theory constructivism and theoretical re-formulation of the data [14] was possibly at odds with positivist views which may have relied more on current observable facts. There was an intention to engage staff, particularly Indigenous Healthworkers, in data analysis to add important contextual insights. Contractual project timeframes combined with Apunipima staff clinical workloads involving frequent travel to remote communities precluded this process. The grounded theory approach did not clearly set out future implications of the research findings for Apunipima staff.

Qualitative researchers must select from an array of representational styles, those that best fit their research purpose, methods and data [15]. The grounded theory theoretical model was abandoned and data from the three participant sources (family participants, Apunipima staff and men’s focus group) were imported into NVivo. A thematic analysis was undertaken to generate initial codes, search for themes among codes and define and name themes. The key study themes are presented with quotes to share the voices of participants and provide illustration of the findings.

Senior Apunipima researchers and the BOP team subsequently came together in a collaborative workshop to develop a strategy for strengthening processes and ongoing evaluation of the BOP. It was important that recommendations could potentially be adopted within day-to-day program delivery context. A series of brainstorming, sorting and prioritising activities using the themes identified in the study data derived a set of practical recommendations that are presented in the Discussion.

## Results

The data revealed the following key themes: challenging environments for new families and valuing cultural ways, resourcing program delivery, working towards a team approach, negotiating the cultural interface, engaging families, exchanging knowledge through ‘yarning’ (conversation), strengthening the workforce, and seeing health changes in families. Data from Apunipima staff interviews were coded to clearly distinguish between staff involved in program delivery, who were all Indigenous Healthworkers (IHW), and those who provided related services (R-S); as doctors, midwives and maternal and child health nurses, allied health staff and health promotion officers; or program support (P-S) via management and administrative roles.

### The central concern and process

From the data, the key to effective program implementation was the relationships formed between health practitioners and families. Program development and quality improvement was contingent on responsiveness to family needs. Staff members identified the need for respect, empathy and positive language: *“…they [families] know that they have a safe place to talk, because I build that relationship”* (IHW). Mothers are essential partners in the BOP, but a relationship with the whole family was viewed as important. *“The way that it should be done is about building relationships with those families and maintaining them over the 1000 days”* (R-S). Some believed program implementation could be more responsive by valuing and validating decision-making capacities of family members. *“It should be more family led…”* (R-S). An Indigenous Healthworker believed the program was achieving this. *“It just lays it all out well for them to be able to take control of their health and their baby’s [health]…it is important for [families] to have…control over their own health.”*

In the process of evaluating context in the implementation of the BOP two key factors were identified from the data: *challenging environments for new families* and *valuing cultural ways*.

### Challenging environments for new families

The conventional view, that people in Cape York Aboriginal communities have close family and kinship ties, implies that the responsibility of caring for children is shared between parents, grandparents, uncles and aunties. However, two of the three mothers interviewed stated that they did not always have family support in caring for their infants. One said: *“You don’t get any other support from your families.”* The other commented *“Well, I don’t have much family. That’s why I’m making family.”*

Family members believed that the mother’s evacuation to Cairns at 36 weeks, without her immediate family, assumes that her other children will be cared for by her partner and/or extended family members. Men noted that exclusion of the father at the birth of his child could establish a pattern of the absent father; they advocated: “*Involvement from the start [will create] acknowledgement of family members…If [fathers are] involved, support will continue. Involve the man more*.” (Men at focus group). An Indigenous Healthworker agreed *“…the father’s got to be a part of that thing from day one…it’s like you want to be with your wife, your newborn, you’ve got to be a part of this child from the beginning”*.

Household overcrowding challenges some families. Mothers reported that living situations can be distressing.

*“I’m tired because we are in the middle of everything, and it’s really noisy, and there’s just parties here, parties there, and it’s like really loud and straight through us. I don’t know how [baby] coped with it all”* and, *“…it’s hard, it’s pretty noisy, but you’ve got to cope with it.”*

A need to provide support for new mothers is clearly recognised by Indigenous Healthworkers.

*“Everything can be a bit too much … you’ve been out of community [in Cairns] for 4-6 weeks, depending on whether you gave birth on your due date…the stresses of being a new mum again.”* (IHW).

Another Healthworker said *“you can see that families really need it, especially first time mums.”*

### Valuing cultural ways

Because many traditional roles of men (as hunters and providers) have changed, fathers are often expected to take on roles and tasks within the family that were once the domain of women. One health practitioner observed:

*“I think it’s shared roles…nutrition is not all mums’ business. And the stuff around the bathing. This is where we challenge fellas. It might be taboo for you to bath babies, but it’s not taboo for you to get the bath ready…A lot of fathers want to be involved, but they just don’t know how*.*”* (R-S).

An Indigenous staff member reflected, *“I think part of building healthy children is strengthening culture. It’s also a reflection time for the mother too, she is reflecting on her culture too, she is passing it on to baby.”* (P-S).

An Indigenous Healthworker confirmed the importance of a family-centred approach in the BOP. She said the program, *“educates the whole family, so it’s in the household and you are giving the information to the mother and her brothers and sisters or her aunty or grandma or who will be there looking after that baby.”* She continued, *“it’s really good because you use your normal body language and eye language and you can do it in a more culturally appropriate way for your community.”*

Three key enablers of BOP implementation were identified from the data: *resourcing program delivery, working towards a team approach* and *negotiating the cultural interface*.

### Resourcing program delivery

The BOP is funded by the Commonwealth and Queensland governments. The initiative is regarded as a flagship program of Apunipima, however resource limitations across the organisation have meant amendments to program support, including plans for expanded employment. Full implementation of the program across all 11 Apunipima communities remained to be achieved at the time of data collection.

Staff workloads are often high and there are substantial service delivery expectations. Healthworkers are responsible for clinical service in addition to health promotion work,*“…it’s a really great concept, but in other ways there’s a lot of pressure on people to do it now and have results now…”* (R-S). The time commitment for Healthworkers was particularly high during the first year of the program because families are enrolled at their first antenatal visit and early scheduled visits are close together. The projected ideal caseload is 25 families but one Indigenous Healthworker reflected, “*In [community name] about 16 or 17 and that is ante and post-natal, and in Cairns there is about 12 on the list.*” A community-based Indigenous Healthworker said *“…we definitely need more Healthworkers on the ground”.*

The program included one full-time position to provide ‘on-the-ground’ training and support to BOP Healthworkers across nine communities. Field support was regarded as an important adjunct to formal BOP Healthworker training. Managers and Healthworkers urged the need for allocation of more resources to heathworker support, however, the high cost of bringing staff together over long distances for formal training blocks resulted in a reduction from quarterly to bi-annual face-to-face training workshops.

### Working towards a team approach

The BOP arose from Apunipima’s strategic decision to realign maternal and child health into a family-centred, Healthworker-led service.

There was divided opinion about involvement of Healthworkers in development of the program. Whilst one staff member believed that the BOP manual was *“…literally dumped…on the Healthworkers, implement it, and own it”* (P-S)*,* an Indigenous Healthworker said *“from the time that I first started, Healthworkers have a real good influence on how the [program] is delivered and how the education…is given to communities.”*

The focus on Healthworker leadership meant that some nurses and midwives felt excluded. A health practitioner said it seemed like, *“they didn't want the RNs [Registered Nurses] involved.”* (R-S). An Indigenous Healthworker said there was a *“big divide between a Healthworker and nurses…even though we don’t have a university degree, we have all this other knowledge that probably can’t be written on paper.”*

A quality improvement approach and new ideas resulted in evolution of the program’s structure and content. Indigenous Healthworkers and midwives reported that their work roles became more clearly defined. A health practitioner commented:

*“We know that it is Healthworker-led…If the Healthworker is struggling and wants assistance, then yeah, they can ask and we can do that…”* (R-S).

A manager acknowledged early confusion over professional roles and reported the steps made to address the concern. *“When a new Healthworker comes on, the midwife is part of that learning journey, so even though it’s…being led by the Healthworker through the cultural expertise, and the midwife as the expert in the clinical…we are walking together.”* (P-S).

BOP implementation has provided an opportunity for knowledge exchange between Indigenous Healthworkers and other health practitioners. A comment by one Apunipima staff member exemplified openness to hearing new ideas,

*“It is an opportunity for us to learn from each other without having to reinvent the wheel…someone within the group may have a suggestion that might knock our socks off and make a real improvement to how we do things.”* (P-S).

Some identified the future challenges of the program and delivery of its full potential. *“I think because the program is in its infancy. There is a lot of room for improvement and there are areas that need to be worked on to ensure that.”* (IHW).

### Negotiating the cultural interface

Apunipima values a strong Indigenous workforce as the way to build culturally-safe relationships with community members and connect them to health care resources.

Ideally, Indigenous Healthworkers can translate and explain Western medical concepts in language more easily understood by family members.

“*If you're going in to a…clinic and you feel scared because your child is sick…people use big words that you're not used to... And if you have the Healthworker that you actually really trust, has been seeing you in your home, knows you, has been knowing you for about a year, and you can look to her and you can say ‘what are they talking about?’”* (P-S).

However, because Indigenous Healthworkers live in the community and are often family members, they may face cultural barriers when working across gender, or kinship relationships. *“…when it is a community person, sometimes they might not be able to speak to or have a lot to do with one side of community”* and*“…you'll find that sometimes in the communities, the female Healthworker may not be allowed culturally to speak to that male or father.”* (P-S). To address these cultural needs, roles are negotiated across program staff members.

Three key strategies of BOP implementation were identified from the data: *engaging families, exchanging knowledge through yarning* and *strengthening the workforce*.

### Engaging families

Initial engagement through the BOP provided a foundation for effective ongoing relationships. A health practitioner explained, *“…to me the engagement part is the most important part. You get that right, everything else is just going to come in”* (R-S) and *“[as] a first time mum…you need to build a relationship with the team that is going to be there for you right through…your pregnancy and then through the upbringing of your child.”* (P-S).

Communication style was cited as important. *“…we use broken English to explain things. I find the BOP gives you more options to talk about what is actually going on with people’s lives, not only the child, but the mum and the dad.”* (IHW). Baby baskets delivered by Healthworkers during family visits are used to engage with families. Most expressed appreciation for the baskets, and found the contents helpful. One mother said, *“The baskets are good, it’s helpful, and I’ve never had anything like that before...”* Another mother said that she did not previously have what she needed for the baby, “*I didn’t have enough chance to get much for the baby when I was down in Cairns [for birth]. It was too much…I couldn’t leave my one year old…”*

It is convenient for some families to be visited at home, especially parents with small children. One mother said, *“Yes, [home visiting] it’s helpful, it saves me going all the way up [to the clinic]...because sometimes I don’t have the time to go.”* Other families prefer to meet the Healthworker outside their house. One Indigenous Healthworker explained, *“We can have it [home visit] outside on the veranda. I wait for them to tell me where they want it to happen…”*. When families *“are more comfortable, they are more likely to open up to you. I find with the BOP that people are inviting us in.”* (IHW).

### Exchanging knowledge through ‘yarning’ (conversation)

Family members noted that knowledge exchange through the program was helpful. One mother agreed that she was able to talk about issues that concerned her and understood the response provided. Indigenous Healthworkers reported that yarning about health and wellbeing topics is best done in the context of a relationship. The preference for having the same Healthworker through a woman’s pregnancy and post-natal period was noted by one mother, “*Yes, I rather stick to one*.”

The BOP manual summarises the content of yarning topics in order to support consistent delivery across Cape York communities. Some Healthworkers discuss the topics in a prescribed order, while others use them according to the needs of the family. Some were reluctant to talk about sensitive topics such as family violence or sexually transmitted infections. *“The workers based in the community, they…were just like no, I don’t feel comfortable with talking about this”* (R-S). However, a senior Indigenous Healthworker, when asked if there were any health promotion topics she felt she couldn’t raise with families, said *“No, I’m quite confident”.*

Sensitive yarning topics were developed and presented via educational videos. A Healthworker said, *“I use the iPad if the lady is real quiet and doesn't talk much…so I just use the visual so she doesn't have to say anything.”* Another staff member said, *“…it [the video] took the pressure off raising and having those conversations…even to raise that topic.”* (P-S).

One Indigenous Healthworker believed that the yarning topics made a positive difference. She reported that a mother *“said ‘you know I’ve never really had education like this before’”* and *“…some of the ladies that we do education around, they kind of nod their head and say ‘oh we didn’t know that’.”*

### Strengthening the workforce

Apunipima’s strategies to strengthen program workforce included training and supporting Healthworkers with a commitment to recruiting new positions from Cape York communities. A health practitioner observed, *“*…*the capacity of Healthworkers is different, so some will need more support and maybe just reminding*.” (P-S). Continuing investment in Healthworker support and training has been regarded as critical to the ongoing successful delivery of the program.

There was also an opportunity to promote coordination between the BOP Healthworkers and the Apunipima Men’s Health Team. An Indigenous Healthworker said, “*I just believe you need to work together for this to succeed.”* A health practitioner agreed *“…it would be a really good program where male Healthworkers were working with the fathers through pregnancy, for a thousand days [and] could become very much involved.”* (P-S).

Early formative outcomes identified from the data of BOP implementation were related to *seeing health changes in families*.

### Seeing health changes in families

The program promoted good health through behaviours such as quitting smoking and reducing consumption of alcohol. *“People will come up to me in community and say that they (don't) smoke any more, or I’ll hear good feedback from other family members”* (IHW). A mother reported her achievement in giving up smoking and drinking, *“…both of us used to be bad at smoking and drinking…but we just gave it up.”*

Healthworkers reported a reduced risk of families engaging with the Department of Child Safety because of the support provided by the BOP.

*“With help of the Baby One Program and the support network of the midwives and the Healthworkers, we’ve actually stopped a lot of that happening. Because we say to Child Safety, ‘oh no, they’re engaged in this parenting program, this is a parenting visiting program, this is what we do every fortnight.’ I tell them ‘we'll come and see this girl.’ So there’s been lots of good outcomes like that.” *(IHW).

Family members also reported feeling more comfortable at their local clinic, visiting the clinic more often and better engagement with clinic staff. A mother noted that she saw the Healthworker “*a lot more*” than for her previous children, and that she liked the Healthworker visits and involvement of the midwives.

A program support staff member concluded that,

*“There is the capability of this program to be a source of pride for Cape York Healthworkers and Apunipima, and that relates to obviously their having a positive impact on the families that they’re supporting and delivering health care to…and that’s the bottom line.”* (P-S).

## Discussion

The BOP was developed by health practitioners and Healthworkers in Apunipima’s family health team. As is common with many health promotion programs, it was based on evidence from previous program evaluations [16–18] in addition to past experience, practical local knowledge and intuition about what might work [19]. The intention of the study was to explore the key features of early implementation of the BOP and especially the challenges. It looked at how health providers connect with families and how well the program responded to practical family and cultural issues associated with pregnancy and early childhood in ways that were culturally and professionally proficient.

The study confirmed that the central factor in program implementation is the quality of the relationship developed between health practitioners, especially Healthworkers, and families. Family members are encouraged to participate in decisions, ask questions and voice their opinions and concerns. Such relationships value and support family autonomy [20]. Relating in this manner brings an obligation to respect diversity and family choices, whilst endeavouring to meet families’ expectations of a primary healthcare service [21]. As such, the BOP aims to build capacity to respond respectfully, skilfully and flexibly to the day-to-day health needs of families.

The BOP’s family-centred approach focuses on health promotion according to the principles of choice, participation and self-determination [22]. Apunipima’s emphasis on accessible, culturally-appropriate, Indigenous Healthworker-led care aligns closely with the priorities of the Australian National Aboriginal and Torres Strait Islander Health Plan 2013–2023 [23]. The program complements and strengthens existing maternal and child health services in Cape York, with greater investment in the Indigenous health workforce. The long-term aim is to provide a sustainable public health strategy to promote family health and social and emotional wellbeing [5].

Past colonisation and assimilation policies have greatly disrupted traditional Indigenous Australian cultures. For many Cape York families, disadvantage is characterised by high levels of unemployment, food insecurity, poor housing, limited education opportunities and geographical remoteness. It is within such environments that Apunipima staff strive to meet the considerable logistical demands of delivering the BOP using ways that value and respect local cultural beliefs and practices. And here, health systems could certainly do more to promote family unity. While family and kinship ties are fundamental to Indigenous societies and underpin delivery of key maternal and child health services, it cannot be assumed that everyone has immediate family support at critical moments in their lives [24]. Government resources are available to fund a support person to accompany a first-time mother to Cairns for birth, but not for subsequent non-complex pregnancies: low incomes preclude most Indigenous families from self-funding subsequent trips. Consequently, many Cape York babies are born in Cairns while fathers and siblings remain in community.

The management and facilitation of such a program must evolve in response to changing circumstances and an embedded quality improvement system is an essential component. Internationally, program leadership by well-trained Indigenous Healthworkers has proven to be effective [5, 9, 25]. The training and employment of Indigenous Healthworkers builds human capital in communities. The degree to which health practitioners are able to engage with families and communities is founded on their knowledge of community, experience in remote settings and cultural background. Importantly, the inclusion of Indigenous knowledge and practice is fundamental to the effectiveness and sustainability of Indigenous health care programs [26]. In the BOP, critical factors were continued investment in recruiting, training and supporting Healthworkers and promoting Indigenous knowledge exchange between practitioners.

There were limitations in this study. They included difficulties in recruiting family members for interviews, and scarceness of research resources that precluded necessary travel to remote communities. Opportunities to recruit BOP families were restricted to the time when expectant mothers were transferred to Cairns to await childbirth. Apunipima staff who had existing relationships with families interviewed family members. These staff were not all experienced interviewers, however, we believe this strategy optimised participant safety and enabled efficient use of scarce resources. Given the study focus on program implementation, the combination of data from family members and other participants was considered sufficient for data saturation.

A further limitation of this study was that, on this occasion, the grounded theory approach for development of the theoretical model was not supported by Apunipima staff as an accurate representation of their experience. The focus of interview questions was across an 18-month developmental period in a rapidly changing practice environment, and the theoretical model was perceived not to reflect current practice. Apunipima staff members were unclear about how the model could be used to inform further practice improvements. According to Glaser [27], grounded theory research is a many-faceted approach requiring considerable time in addition to theoretical sensitivity to move from the data to the theory and back. Fit and relevance of the theory are two essential factors, and it must work. In the field of applied health research, limitations of funders and ethics committees may constrain engagement in open-ended sampling processes and analytical creativity required by grounded theory, leaving consideration of alternative research methods as a solution. Studies do not always play out as expected; in this way it is important to recognise a digression from the path and be transparent about it. However, the themes identified by the study were valuable for internal organisational reflection. The outcome was a subsequent inclusive and creative process that produced a set of practical recommendations, giving the BOP staff a strong sense of completion.

Recommendations from the study fell into four categories. Under the first category, ‘community consultation and program promotion’, the recommendations were: 1. Ensure that participating communities are engaged in comprehensive and ongoing consultation. Care should be taken to include community members, particularly Elders, local councils and health action teams, and 2. Raise community awareness of the program using the tenets of social marketing and principles of program branding in a four-step process (i) Planning and strategy development (ii) Create and test concepts, messages and materials (iii) Implementation (iv) Assess effectiveness and make refinements. In the second category, ‘responsive health services’, the recommendation stated that health services should be responsive to family needs; for example, more could be done to promote family unity by supporting fathers and other children to travel with the mother for the birth of a new family member. In the third category, ‘professional development’, recommendations were: 1. Continue essential up-skilling, education and field support for Healthworkers, midwives and maternal and child health nurses, and 2. Support and encourage knowledge exchange between Healthworkers, midwives and maternal and child health nurses. New program staff should receive complete orientation that highlights this critical aspect of the program. The final category included recommendations concerning formal processes for ongoing program evaluation. They were: 1. Identify appropriate health outcome indicators, data collection methods and obtain formal ethical approvals and informed consent from families for continuous monitoring of program effectiveness and 2. Develop and embed program evaluation components to measure family satisfaction with service provision at identified time points across family engagement with the program. The substance of such recommendations may be transferrable to other maternal and child health innovations designed for Aboriginal and Torres Strait Islander families*.*

The development and implementation of the BOP has presented substantial logistic, financial and practical challenges. In contrast to international Indigenous family-centred interventions which have taken a decade to develop [5], this review of the BOP is based on 18 months of developmental implementation. Even when fully developed, other programs have found that expected outcomes had only clearly emerged up to 3 years following birth [5]. The findings of this study suggest that ongoing evaluation of program quality and key outcome indicators is essential for detecting health changes attributable to a program. The lessons learned in this study have enabled a vision for future directions to strengthen Healthworker-led, family-centered maternal and child health care in remote locations. Our review of early program implementation has prompted clear recommendations developed by Apunipima staff to maximise opportunities for giving children the best start to a healthy life. The recommendations for ongoing community consultation and program promotion, responsive health services, professional development and embedded program evaluation processes are likely to be universally relevant to community-based health promoting programs.

## Conclusion

Strengthening family health and wellbeing in remote Indigenous communities is a complex task. This paper describes a model of care designed as a Healthworker-led, family-centred program that has been implemented in very remote Aboriginal communities of Cape York. The program engaged 161 families over an initial 18-month period and continues to grow, engaging more families as women present for their first antenatal check. This research found that the relationships formed between health practitioners and families, and program responsiveness to family health and cultural needs in challenging environments were key to successful implementation. This is maintained by regular Healthworker contact with families aligned with antenatal and child health checks, where Baby Baskets are delivered and ‘yarning’ topics explored. Even though the BOP implementation has presented substantial challenges, it continues to evolve. Healthworker education and training, and knowledge exchange between Healthworkers, midwives and nurses was seen as critical for strengthening the workforce, a team approach and seeing positive health changes in families due to program effectiveness. This is where resources must be gainfully invested for long-term success. Ongoing evaluation of the program, including built-in collection of health outcome indicators, should drive improvements in service delivery and better outcomes for Cape York children and families. The goal is a healthy start to a healthy life.

## Additional files

Additional file 1: ‘Aboriginal & Torres Strait Islander Health Workers’. BOP Evaluation interview guide. (DOCX 20 kb)

Additional file 2: 'Managers and Clinical and Allied Health Staff'. BOP Evaluation interview guide. (DOCX 21 kb)

Additional file 3:  'Mums and family members'. BOP Evaluation intervew/focus groups guide. (DOCX 18 kb)

## Abbreviations

Apunipima: Apunipima Cape York health council
BOP: Baby One Program
IHW: Indigenous healthworker
NVivo: Qualitative data analysis software
P-S: Study participants who provided program support to the BOP via management and administrative roles
R-S: Study participants who provided related services to the BOP as doctors, midwives, maternal and child health nurses, allied health staff and health promotion officers
