# Supplementary material for: Implementing the Baby One Program: a qualitative evaluation of family-centred child health promotion in remote Australian Aboriginal communities
Source: BMC Pregnancy Childbirth. 2018 Mar 24;18:73. doi: 10.1186/s12884-018-1711-7 (PMC5866524; doi:10.1186/s12884-018-1711-7)
Supplement: Supplementary file 1 — ‘Aboriginal & Torres Strait Islander Health Workers’. BOP Evaluation interview guide. (DOCX 20 kb) [file 12884_2018_1711_MOESM1_ESM.docx]

**BOP EVALUATION INTERVIEW SCHEDULE 1:**

***BOP Aboriginal & Torres Strait Islander Health Workers***

- Which community/communities do you provide the BOP in?
- Do you practice other duties aside from delivering the BOP?
- When did you first become involved in the BOP and what is your understanding of the BOP generally?
- How many Families/Mums/Babies do you work with in the BOP?
- When you are working with families, can you tell me how you usually get started and how you deliver the BOP to them?

***Home visits:***

- Do you do many of the BOP visits in the home?
- If not, where do you have the visits and why?
- What do you think about the idea of having BOP visits in the home?
  - - What do you **enjoy most** about visiting mums in their homes?
    - Do you think the dads are interested in the program?
    - Who usually joins in with the visit?
    - Who do you think should be there?
    - What do you find **most challenging** about visits in the home?
    - What do you find **most useful** about the home visits?
    - What do you find is the **least useful** about visiting families in their home?
- Do you think the BOP families like/would like home visits? If not, why not?

***Yarning topics:***

Of the 37 Yarning topics listed in the BOP Manual:

- What yarning topics are usually easy to talk about?
- Are any of the yarning topics harder to talk about than others?
- Where some yarning topics are hard to talk about, do you have another way of covering it with the family?
- Are there any yarning topics that you feel you cannot cover? If yes, why do you feel like you can’t talk about them? Do you think it would be better for a different person to cover those topics?

***Engagement (& family-centred approach):***

- Do you think the mums engage better about their pregnancy because of the BOP? If so, can you say how and why you think so?
- Do you think the BOP helps to improve your connection with the mum and family?
- Do you usually get to know other family members, as well as the mum?
- Do you think it helps improve the mum’s engagement with the clinic, midwives and doctors?
- What about other family members? Is it useful for engaging the dads?

***Health worker Education:***

- Can you tell me about the health worker training you attend as part of the BOP?
- How does the Cairns BOP training strengthen the way you deliver the BOP?
- How well do you feel your training equips you to talk about all the BOP yarning topics?
- Are there things you need to know to do your job well that are not covered in the BOP training?
- What are your suggestions for improving the BOP health worker training?

***Health worker led program:***

- What kind of input do you have in the development and shaping of the BOP program and how it’s delivered?
- What do you think are the strengths of the program for health workers?
- How are you able to demonstrate leadership in you work with the BOP?

***Social & Emotional Well-Being:***

- What are the types of things that you hope the BOP can help improve for mums, babies and their families in Cape York?
- How do you think you will be able to tell if the program is working well and making improvements?
- What things do you think could stop the program from working well?
- Can you give an example/examples of when you’ve felt that (as a BOP health worker) you’ve been able to make a positive difference for a mum, her baby and/or her family?
- Have you come across family problems that you’d like to help with, but found that you weren’t able to?

***General questions:***

- Do you have any worries about the BOP?

About how it’s developed?

About how it’s delivered?

About how it’s managed?

- Is there anything missing from the BOP?
- Can you think of ways it could be made better?
- Is there anything else you’d like to talk about today that will benefit the BOP?
