# Supplementary material for: Implementing the Baby One Program: a qualitative evaluation of family-centred child health promotion in remote Australian Aboriginal communities
Source: BMC Pregnancy Childbirth. 2018 Mar 24;18:73. doi: 10.1186/s12884-018-1711-7 (PMC5866524; doi:10.1186/s12884-018-1711-7)
Supplement: Supplementary file 2 — 'Managers and Clinical and Allied Health Staff'. BOP Evaluation interview guide. (DOCX 21 kb) [file 12884_2018_1711_MOESM2_ESM.docx]

**BOP EVALUATION INTERVIEW SCHEDULE 3:**

***Managers and Clinical and Allied Health Staff***

- Do you have a role in the BOP?

If so, when did you first become involved in the BOP?

In which communities?

- What is your understanding of the BOP generally?
- Can you tell me about any positive changes you are aware of in maternal and child health care since the BOP started in July 2014?

***Home visits:***

- Do you have thoughts about delivering the BOP with home visits in the communities?
- Do you think the BOP families like/would like home visits? If not, why not?

***Yarning topics:***

- What is your knowledge about the yarning topics included in the BOP?
- There are 37 topics for the Health Workers to cover with the families? Do you have any thoughts on the content of the yarning topics you would like to share?
- Are there any yarning topics you think are missing?
- Are there any yarning topics you don’t think should be included?
- Do you have any suggestions to improve the yarning topics?

***Engagement (& family-centred approach):***

- Do you think the mums engage with health around their pregnancy because of the BOP? If so, can you say how and why you think so?
- Do you have any examples of how the BOP has improved a mum’s engagement with the clinic, midwives and doctors?
- Can you suggest ways to help improve mums, dads and families engagement with BOP?
- How well do you think other community programs & organisations/bodies are engaged with the BOP? (Women’s Shelters, HATs, Community Councils, Cape York Partnerships, Act for Kids, Child Safety).
- How important is communication and engagement between the BOP and other community programs?
- Are there ways you think communication and engagement with those programs could be improved?

***Health Worker Education:***

- Do you think the Health Workers are equipped with the knowledge and skills to deliver the BOP?
- Do you have any knowledge about the BOP Health Worker training that’s held in Cairns?

If yes, can you tell me your thoughts about the training?

Do you have any suggestions for improving the BOP Health Worker training?

***Health Worker leadership & program development:***

- How is effective Health Worker leadership demonstrated in the BOP?
- What do you think are the strengths of a Health Worker led approach?
- What type of input have you had into the development and shaping of the BOP program and how it’s delivered?
- If none, why haven’t you had input? Would you have like to have input into the BOP program? If so, what would you like to contribute?
- Have you had the opportunity to provide input to the BOP Manual?
- Do you feel like you have the ongoing opportunity to influence the direction of BOP?

***Social & Emotional Well-Being (SEWB):***

- What SEWB outcomes do you hope the BOP will improve for mums, dads, babies and their families?
- How do you think the BOP is improving the wellbeing of mums, dads, babies and their families in Cape York?
- Can you give an example/examples of when you’ve felt that the BOP has made a positive difference for a mum, her baby and/or her family?
- Do you have any ideas on how SEWB improvements could be measured?
- Have you come across family problems that you don’t think the BOP would be able to help with?
- How do you think BOP can improve service delivery for SEWB?

***Best Practice and iPad systems:***

- Do you know about the iPads the Health Workers use in the BOP?

Would you like to share anything about the iPads?

- Do you have anything you’d like to share about recording BOP activities in Best Practice?
- Can you suggest ways to improve the electronic patient record systems?

***General questions:***

- How aware and supportive do you think other clinical service providers in communities (RFDS, QH) are of the BOP?
- Have you noticed or heard about any duplication of services in relation to the BOP?
- Is there anything else you’d like to talk about today that will benefit the BOP?
