# Supplementary material for: Implementing the Baby One Program: a qualitative evaluation of family-centred child health promotion in remote Australian Aboriginal communities
Source: BMC Pregnancy Childbirth. 2018 Mar 24;18:73. doi: 10.1186/s12884-018-1711-7 (PMC5866524; doi:10.1186/s12884-018-1711-7)
Supplement: Supplementary file 3 — 'Mums and family members'. BOP Evaluation intervew/focus groups guide. (DOCX 18 kb) [file 12884_2018_1711_MOESM3_ESM.docx]

**BOP EVALUATION INTERVIEW/FOCUS GROUPS SCHEDULE 2:**

***Mums and family members***

- When did you first become involved in the BOP?
- What is your understanding of the BOP generally?
- Which community do you live in and receive the BOP?
- Do you have any other children that you care for?

***Home visits:***

- Does the Health Worker visit you at your home to talk about the BOP?

If not at home, where do you have the visits (and why)?

- How many BOP visits have you had at home? (what stage of pregnancy or how old is Baby?)
- When the Health Worker comes to visit you, can you tell me how the visit usually gets started?
  - - Who usually joins in with the visit?
    - Who do you think should be there?
    - What do you find **most challenging** about visits in the home?
    - What do you find **most useful** about the home visits?
- What do you find is the **least useful** about the BOP visiting your family at home?
- What suggestions do you have for improving the BOP visits?

***Yarning topics:***

- A big part of the BOP is to talk to families about ways to improve health. The Health Worker is guided by a list of topics to cover at each visit. Can you talk about your experience with each of the topics?
- Which yarning topics have been covered with you so far?
- What yarning topics are usually easy to talk about?
- Are any of the yarning topics harder to talk about than others? Which ones?
- Do you get to talk about the information you receive from BOP with other people in your family or community? Who do you share information with?
- Can you suggest any ideas to improve the yarning topics?
- What is the biggest change you have experienced as a result of receiving the BOP in your community? (this might be change to how you parent, change to the way you view MCH Services or the clinic).

***Health Information & iPads***

- How useful is the information the BOP Health Workers give you? Is it interesting? Easy to understand?
- Can you tell me about some of the new things you’ve learned from the BOP (that you didn’t know before)?
- What things do you do differently because of what the BOP Health Workers talk to you about?
- What do you think are the most important things you can do to keep yourself healthy and happy while you are pregnant?
- What do you think are the most important things you can do to keep your children healthy?
- Have you used the BOP iPad?
- If yes, what did you use it for?
- Do you think the BOP iPads are useful? What are they useful for?

***Engagement (& family-centred approach):***

- Do the BOP Health Workers remind you about your visits at the clinic with the midwife or doctor?
- Does being in the BOP encourage you to go to the clinic for health care in pregnancy?
- Does being in the BOP encourage you to take your children to the clinic for check-ups?
- Do you always go to clinic visits when they are due? (If not, why?)
- Do you think that baby’s dad is interested in the program?
- How do you think the BOP should try to involve family members in your pregnancy health care?
- How do you think the BOP should try to involve family members in health care for your baby?

***Health Worker led program:***

- Where do you get most of your health information during pregnancy?
- Where do you get most of your health information about children’s health?
- Who are the most important people that provide health care for you while you’re pregnant?
- Who provides most of the health care for your children?
- How do you feel about having a program like BOP that is run by Health Workers?
- How do you feel about having a program like BOP that is delivered by Apunipima?

***Social & Emotional Well-Being:***

- Does being in BOP support you and your family to be strong in culture and spirit?
- How do you think the BOP Health Worker can support you when you need help with health or family problems? Please provide examples.
- Does the BOP Health Worker talk to you about smoking and drinking during pregnancy/around children? Have you made any changes in your home about smoking or drinking since you’ve been in the BOP?

***General questions:***

- Do you feel as though you are well supported by the BOP and the BOP Health Worker?
- Is there anything missing from the BOP? (eg. Male Health Workers, Preconception/contraception…)
- Is there anything else about the BOP that you’d like to talk about today?
